# Age determination of common bottlenose dolphins (*Tursiops truncatus*) using dental radiography pulp:tooth area ratio measurements

**DOI:** 10.1371/journal.pone.0242273

**Published:** 2020-11-20

**Authors:** Jean M. Herrman, Jeanine S. Morey, Ryan Takeshita, Sylvain De Guise, Randall S. Wells, Wayne McFee, Todd Speakman, Forrest Townsend, Cynthia R. Smith, Teresa Rowles, Lori Schwacke

**Affiliations:** 1 Companion Animal Dental Services, Bolton, Connecticut, United States of America; 2 National Marine Mammal Foundation, San Diego, California, United States of America; 3 Department of Pathobiology and Veterinary Science, University of Connecticut, Storrs, Connecticut, United States of America; 4 Chicago Zoological Society’s Sarasota Dolphin Research Program, c/o Mote Marine Laboratory, Sarasota, Florida, United States of America; 5 NOAA Center for Coastal Environmental Health and Biomolecular Research, Charleston, SC, United States of America; 6 Bayside Hospital for Animals, Fort Walton Beach, Florida, United States of America; 7 Office of Protected Resources, National Marine Fisheries Service, National Oceanic and Atmospheric Administration, Silver Spring, Maryland, United States of America; Animal Health Centre, CANADA

## Abstract

Age is an important parameter to better understand wildlife populations, and is especially relevant for interpreting data for fecundity, health, and survival assessments. Estimating ages for marine mammals presents a particular challenge due to the environment they inhabit: accessibility is limited and, when temporarily restrained for assessment, the window of opportunity for data collection is relatively short. For wild dolphins, researchers have described a variety of age-determination techniques, but the gold-standard relies upon photo-identification to establish individual observational life histories from birth. However, there are few populations with such long-term data sets, therefore alternative techniques for age estimation are required for individual animals without a known birth period. While there are a variety of methods to estimate ages, each involves some combination of drawbacks, including a lack of precision across all ages, weeks-to-months of analysis time, logistical concerns for field applications, and/or novel techniques still in early development and validation. Here, we describe a non-invasive field technique to determine the age of small cetaceans using periapical dental radiography and subsequent measurement of pulp:tooth area ratios. The technique has been successfully applied for bottlenose dolphins briefly restrained during capture-release heath assessments in various locations in the Gulf of Mexico. Based on our comparisons of dental radiography data to life history ages, the pulp:tooth area ratio method can reliably provide same-day estimates for ages of dolphins up to about 10 years old.

## Introduction

For developing a better understanding of wildlife populations, it is critical, but often difficult, to accurately establish an animal’s age. Reliable age estimates are necessary for interpretation and application of biological and contaminant data, population demographics, reproductive status, and necropsy results. Determination of chronological age is the ultimate goal, but at a minimum, age class approximation (e.g., calf, subadult, adult, geriatric) is vital to research and for potential veterinary assessments and care. Age determination for marine mammals can be particularly difficult as a result of their cryptic nature and the remote locations many species inhabit. Thus, researchers have developed a variety of methods in an attempt to estimate age in marine mammals, each giving varying accuracy and precision. These techniques include, but are not limited to, long term photographic-identification (photo-ID) monitoring in live animals [1], morphometric comparisons in live and dead animals [2–4], dentinal growth layer groups from extracted teeth (GLGs) [1, 3, 5, 6], bone GLGs in carcasses [7, 8], skeletal ossification live dolphins [9], bone density [10–12], micro-CT scanning [13], radiometric aging in auditory bullae from carcasses [14], ear plug layers in mysticete carcasses [15], aspartic acid racemization in carcasses [16–18], baleen length or radiocarbon dating in carcasses [19, 20], bomb radiocarbon dating in extracted teeth for animals alive in the 1960s [18, 21], telomere sequence analysis in live animals [22–24], DNA methylation analysis in live animals [25], and fatty acid signatures in live or dead animals [26–29]. Unfortunately, many of the aforementioned techniques are inapplicable for use in studies with living dolphins, are unsuitable to be used in field studies, and/or provide poor precision.

In wild dolphins, the most accurate method for determining age is through observational life history evidence, collected through long-term photo-ID studies in which individual animals are initially observed within the first weeks or months of life (hereafter referred to as life history). However, this information is not available for most populations and individuals. Length, and to a lesser extent mass or maximum girth, is often used as a proxy to assign age class in common bottlenose dolphins (*Tursiops truncatus*). However, growth can be influenced by nutrition, habitat, overall health, age, sex, or season, and measurements are less informative once dolphins approach asymptotic length (i.e., growth rate approaches zero) [2, 4, 30]. Given that dolphins reach asymptotic length at approximately 15 years old [31] and are long-lived animals that can have lifespans > 60 years [32], using morphometrics as a proxy for age can be restrictive for studies with older demographics.

Some researchers have attempted to use telomere length to estimate age in dolphins, but this approach is confounded by interfering sequences, which render the method ineffectual [24]. However, another molecular method has been more successful, in which initial research involving a sample of known-age dolphins has identified relationships between epigenetic modifications in age-responsive genes and chronological age in bottlenose dolphins [25]. Measurements of bone density alone, or in conjunction with length, were recently found to poorly correlate to age in bottlenose dolphins [10], despite earlier studies reporting reasonable correlation between age and bone density in younger dolphins [11, 12]. Most recently in dolphins managed under human care, analysis of skeletal ossification using pectoral flipper radiographs was shown to accurately estimate age, with variability increasing with age, using a relatively non-invasive approach [9]. However, the technique has not yet been validated in wild animals, nor directly compared to other age estimation techniques.

Counting tooth GLGs in longitudinal sections of teeth is a long-standing and widely used method for establishing chronological age in dolphins [1, 2, 4, 33–36]. Although the definition of GLG is of structures and not time, generally the implied time frame of the structural GLG is deposited annually for most cetaceans [37]. In mammals, the annual deposition of secondary dentin in the tooth is a consistent, life-long process and occurs at a constant rate in association with age [1, 38, 39]. However, as with body length, the resolution of tooth GLGs is more challenging in older animals because dentin layers often decrease in size and may overlap with increasing age [40], and in younger animals the counting of accessory layers can lead to an overestimation of age in dolphins [1]. In addition, error in these measurements can be introduced through extraction of a non-vital (dead) tooth, sectioning and staining variability, and/or variation in the interpretation of layers among replicate readings or between personnel [1], although recent data suggests high concordance among experienced personnel [41, 42]. The tooth GLG method is invasive as it requires extraction of a tooth and is logistically challenging in the field. Given the constraints associated with the techniques described above, it is essential to continue to develop and refine techniques for age estimation that are as accurate as possible, practical in field settings for live animals, and sympathetic to the health and safety of individual animals.

Another approach to age determination in mammals focuses on the age-associated changes in dental pulp, a loose connective tissue encased within the annual layers of dentin [reviewed in 38]. In humans, the dental pulp narrows with age as additional layers of dentin are deposited by odontoblasts that line the outer layer of the pulp [43]. Dentin deposition continues with age, as long as a tooth is viable, but the rate at which it is deposited slows with age, presumably due to the decreased number of odontoblasts with age [38]. This narrowing continues throughout a lifetime, regardless of health status in vital teeth [38, 39, 44]. Pulp length and width appears to be unaffected by severe dental attrition, based on studies in diverse extant populations and ancient human skeletal remains [45–47]. Thus, Kvaal et al. (1995) utilized standard periapical dental radiography on human patients to propose the first promising quantitative pulpal measurement method, wherein ages estimated by tooth and pulp length or width ratios showed good correlation to known ages [45].

A decade later, Cameriere et al. (2009) improved upon this method by using periapical radiology to measure the ratio of the pulp area to the tooth area (pulp:tooth area ratios) [43, 48]. This method was successfully applied to European skeletal remains [44, 49–51] and has since been validated as an accurate method for aging in diverse human populations, with both skeletal remains and living individuals [52–54]. This straightforward and rapid analysis is relatively objective: by using digital images of the tooth and pulp cavity to determine a ratio of areas, the technique is not sensitive to variations in the angle of image capture. The analysis does not require highly specialized software or specially trained personnel beyond basic dental X-ray experience. Recently, Cameriere’s method was applied in a wildlife species to accurately estimate age within 6 months of known ages in managed African lions (*Panthera leo*) from 3 to 13 years old using both live animals and skeletal remains [55].

Here, the application of digital periapical radiography pulp:tooth area ratios during health assessments of wild bottlenose dolphins in the northern Gulf of Mexico is described. In this study, Cameriere’s method is applied to estimate age from dental periapical radiographs obtained in the field from wild animals with known ages established through life history observations. Additional comparisons for live and dead, stranded dolphins were made between age estimates from pulp:tooth area ratios and dentinal GLGs or length. The resultant model using pulp:tooth area ratios can be applied to radiographs from animals of unknown age to improve the interpretation of data obtained during capture-release health assessments and in rescue and rehabilitation settings by quickly and accurately estimating ages of calves and subadults using this minimally-invasive methodology.

## Materials and methods

Dental radiographs from wild common bottlenose dolphins were collected during capture-release health assessments, conducted in and around the waters of Sarasota Bay, FL (SB) during 2013–2016 (n = 38), Barataria Bay, LA (BB) during 2013–2014 and 2016–2018 (n = 134), and Mississippi Sound, MS (MS) in 2013 (n = 10) (Table 1). Health assessments were carried out under NMFS Scientific Research Permit Nos. 932-1905/MA-009526 or 18786 issued to Dr. Teresa Rowles, or NMFS Scientific Research Permit No. 15543 issued to Dr. Randall Wells. Protocols were reviewed and approved by Mote Marine Laboratory and NOAA Institutional Animal Care and Use Committees for Sarasota Bay and Barataria Bay, respectively. Methods for the temporary restraint and release of wild dolphins have been previously described [56, 57]. Briefly, a seine net was deployed around a group of one to four dolphins, encircling the animals until they were manually restrained by experienced handlers. Dolphins were continuously monitored by veterinary staff and most health assessment processing took place in the water; however, some dolphins were brought onboard a specialized processing vessel for a minimal amount of time (< 45 min). Additional radiographs were obtained from carcasses in the Barataria Bay region recovered from 2011 to 2014 by regional stranding response organizations. The heads were retained, frozen and radiographed at a later date. A random subset of the dead dolphins (n = 23 of approximately 125 total) was included for aging analysis (no specific selection criteria were applied).

**Table 1:** Sample sizes for each age determination technique by sample group.

Group	Dental Radiograph	Male	Female	ND	GLG Age	LH Age	Length (cm)
**BB**	133	63	70	0	50	9	177–284
**MS**	10	10	0	0	10	0	202–265
**SB**	38	17	21	0	7	31	178–285
**Stranded BB**	23	11	8	4	23	0	97–284

BB: Barataria Bay, MS: Mississippi Sound, SB: Sarasota Bay, GLG: Dentinal growth layer group analysis, LH: Observational life history, ND: Data on sex not available.

### Age estimation by life history, GLGs and morphometrics

The dolphin population in SB is well-studied and substantial life history data are available from photo-identification of individuals to confirm chronological age for method validation [32, reviewed in 58]. Likewise, ongoing photo-identification efforts in BB since the *Deepwater Horizon* oil spill have resulted in sufficient information to establish age for many younger animals in that population [58–60]. In these populations, ages have been verified based on photo-ID data collected shortly after birth (i.e., sighted as a neonate with fetal folds present) and therefore accurate within approximately 6 weeks [61, 62]. For GLG determination from a subset of dolphins, a tooth was extracted with local anesthesia (carbocaine 3%) circumferentially injected into the periodontal ligament space [63]. If present, the 15^th^ left mandibular tooth was selected because it has a straight root profile that minimizes multiple curvatures of the tooth, which can interfere with GLG counting [1]. If the 15^th^ left mandibular tooth was missing, the nearest adjacent tooth was selected. After extraction, the tooth was placed into formalin for preservation until sectioning for GLG analysis [1, 4]. Sectioning and GLG examination were performed following the protocols of Hohn et al. [1]. Since length is often used to estimate age class when no other data are available, age classes were assigned for all aging techniques (S1 Table) using the following established criteria [64], calf: < 2 years or < 200 cm; subadult: ≥ 2 years to <10 years or ≥ 200 cm to < 240 cm; adult: ≥ 10 years or ≥ 240 cm.

### Dental radiography

**Fig 1:**
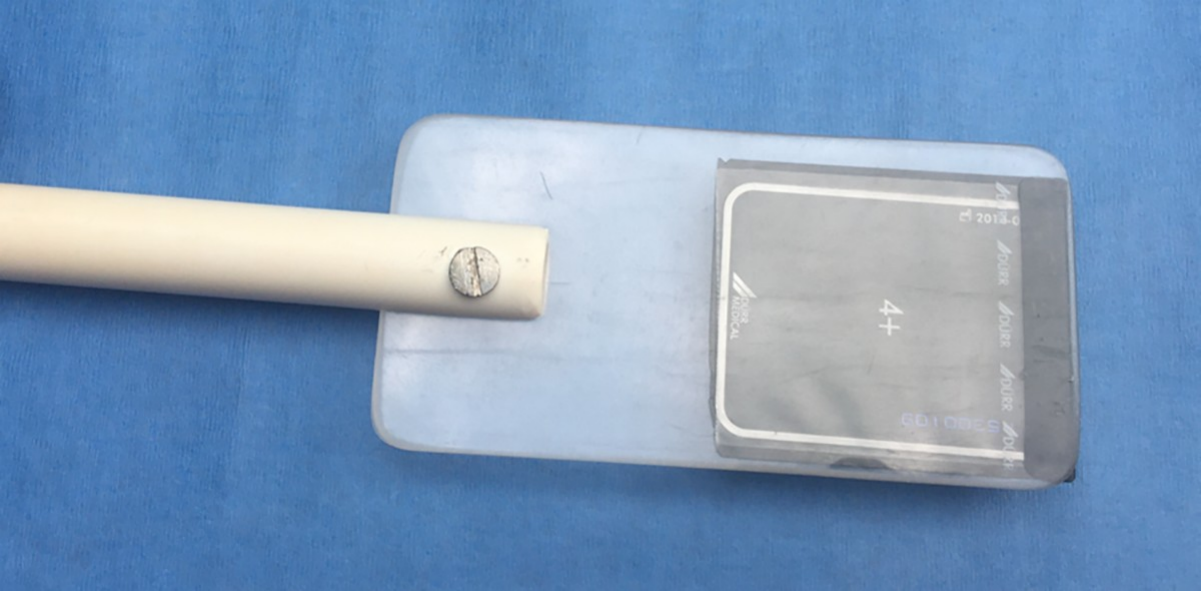
Film holder for dental radiography. A plate holder was manufactured from two appropriately sized translucent plexiglass sheets affixed on the end of a ½ inch PVC pipe. A high-resolution standard dental #4 size digital phosphor plate was encased in a sealed plastic sleeve, inserted between the plexiglass sheets and positioned in the dolphin’s mouth for dental radiographs.

**Fig 2:**
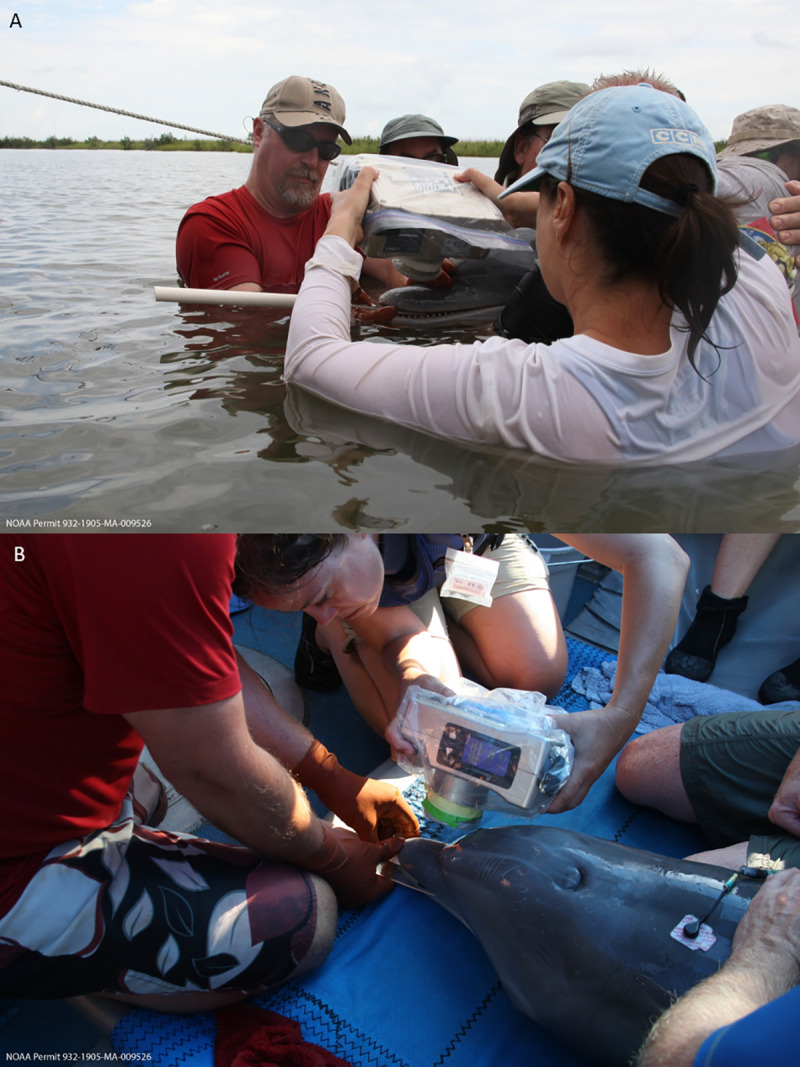
Periapical dental X-ray procedure on wild bottlenose dolphins. During health assessments, water-proofed, portable X-ray equipment was used to collect lateral views of the maxillary or mandibular teeth, both while dolphins were held in the water (A) or evaluated on a processing vessel (B). The lead handler positioned the film holder, while the two other handlers closest to the head helped minimize side-to-side head movements. The individuals appearing in Fig 2 have given written informed consent (as outlined in PLOS consent form) to publish this picture.

Radiographs were performed in the water or onboard a vessel, as determined by veterinary staff monitoring each dolphin during processing. Dead, stranded animals were radiographed in the same manner as in the field; most stranded animals had been previously frozen and thawed with teeth remaining in situ before intraoral radiography was performed. Digital periapical radiology was performed using a lead-shielded, FDA-approved, hand-held ZEN-PX2 X-ray generator (Genoray America, Inc., CA, USA) with a fixed kVP of 60 at 2 mA for 0.4 seconds. Images were captured with a high-resolution standard dental #4 size digital phosphor plate (Duerr Medical, Vancouver, WA, USA). The phosphor plate was encased in a sealed plastic sleeve (iM3 Inc.) and held in a film holder made of appropriately sized plexiglass sheets affixed on the end of a ½ inch PVC pipe (Fig 1). The primary handler, wearing lead- and latex-free, radiation Attenuator X protective gloves blocking 66% at 60 KVP (Z&Z Medical, Inc., Cedar Falls, IA, USA), opened the dolphin’s mouth slightly and gently inserted the plate into the oral cavity (between the mandibular and maxillary teeth) and allowed the dolphin to close its mouth on the holder. The primary handler gently kept the dolphin’s mouth closed on the holder to maximize the dolphin’s comfort and to minimize movement during the radiography. The film in the holder was positioned intra-orally, roughly perpendicular to the long axis of the teeth with the majority of the film surface within the oral cavity. The radiographer directed the X-ray beam at a bisecting angle for the lateral views of the maxilla or mandible (Fig 2). In general, and unless abnormalities warranted otherwise, maxillary teeth 6–12 (typically mid-maxilla) were targeted for age determination. Beginning with the 2014 health assessments, if a tooth was extracted, it was also radiographed before being fixed for GLG analysis.

Two types of X-ray dosimetry badges were used to monitor incidental radiation exposure of the handlers and radiographer. The primary handler positioning the film into the dolphin’s mouth wore a ring-type dosimetry badge under the radiation attenuated gloves. The primary handler, two other handlers closest to the dolphin’s head, and the radiographer wore clip-on dosimetry badges near their neck when onboard a vessel or on hats when in the water. All badges were wrapped in plastic for water resistance. Badges were obtained from and analyzed by a commercial laboratory (Radiation Detection Company, Georgetown, TX, USA) after each field campaign.

**Fig 3:**
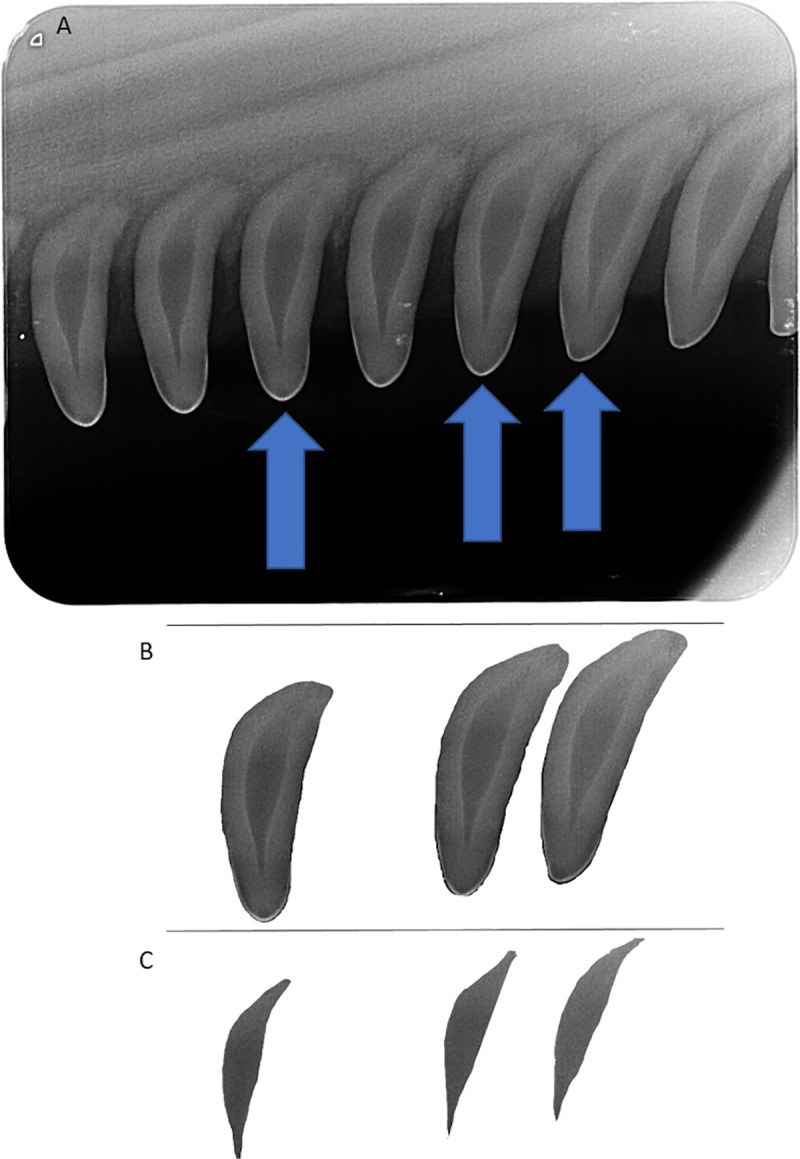
Periapical dental X-ray radiograph from a wild bottlenose dolphin. Three maxillary teeth per individual (A) were selected to calculate the tooth area (B) and pulp area (C) using similar methods to those described by White et al. [55]. Briefly, the lasso and histogram tools in Adobe Photoshop were used to count the number of pixels in each area, then the pulp:tooth area ratio was used to estimate the age of the individual.

Images were scanned and digitized to 251p with a CR7 Vet laser processor (iM3 Inc., Vancouver, WA, USA) using Vet-Exam Intra software (Duerr Medical). Each digitized image typically captured 5–7 teeth, from which three teeth were selected for age determination by the analyst. Visually similar teeth were selected in order to exclude teeth that might have been affected by excessive wear or tooth death. Tooth and pulpal outlines, respectively, were identified in Adobe Photoshop CS6 (Adobe, San Jose, CA, USA) using the standard lasso tool and processed independently as layers. Pixels in each layer were counted by the histogram tool which equated to area measurements for calculation of pulp:tooth area ratios, using similar methods to those described by White et al. [55]. Results from three teeth were averaged to determine the ratio for each dolphin (Fig 3). Approximately 25% (n ≈ 50) of the dental radiographs were reassessed by two additional analysts, including tooth selection and calculation of the pulp:tooth area ratio, to validate the primary analyst’s findings.

### Statistical analysis

All data were analyzed using R (v 4.0.0) [65] through RStudio (v 1.2.5042) [66] and the tidyverse package [67]. To determine the accuracy of pulp:tooth area ratios in estimating age, two approaches were used: 1) a generalized additive model (GAM) was fitted to life history data using a gamma distribution, a log link function, and smoothing using restricted maximum likelihood (REML) estimation with the R packages mgcv and MASS (to calculate the coefficient of variance [CV]) [68–70], and 2) a linear-log model. Root mean square error (RMSE) of the linear-log model was calculated by the leave-one-out cross-validation method, using the caret package [71] to assess predictive power, and root mean square prediction error (RMSPE) was calculated to evaluate the model fit compared to the tooth GLG age estimates.

## Results

**Fig 4:**
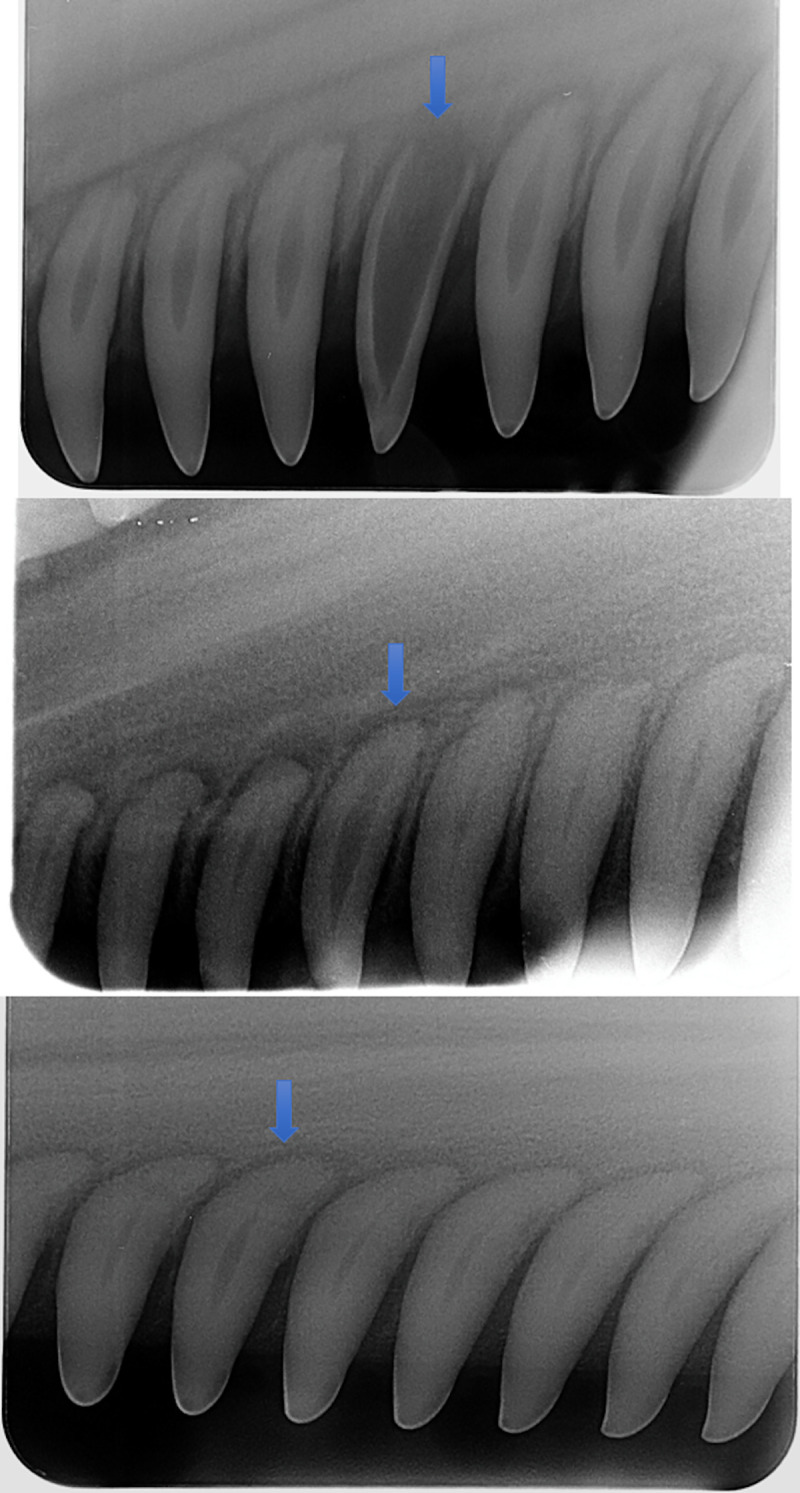
Examples of non-vital teeth. Each radiograph typically captured five to seven teeth, allowing analysts to identify and exclude non-vital (dead) teeth (blue arrows) from the pulp and tooth area calculations, as these teeth no longer accumulate dentin layers as the dolphins age.

Dolphins, under minimal handling and restraint, generally tolerated the digital periapical radiology procedure well, both in the water and onboard the processing vessel during health assessments (Fig 2). The dosimeter badges from the radiographer, lead handler, and the two handlers closest to the dolphins’ heads demonstrated that none of the field personnel were exposed to radiation levels above background. The minimally invasive procedure routinely produced high-quality images suitable to apply Cameriere’s method [43, 48] for estimating age in this small cetacean species (Fig 3). The ability to image multiple teeth on an X-ray film allowed for the elimination of outliers, such as non-vital (dead) teeth with larger pulp that no longer accumulated dentin. Non-vital teeth (dead), both severe and subtle, observed opportunistically in a few dolphins (Fig 4) were excluded from the data analysis for age determination.

**Fig 5:**
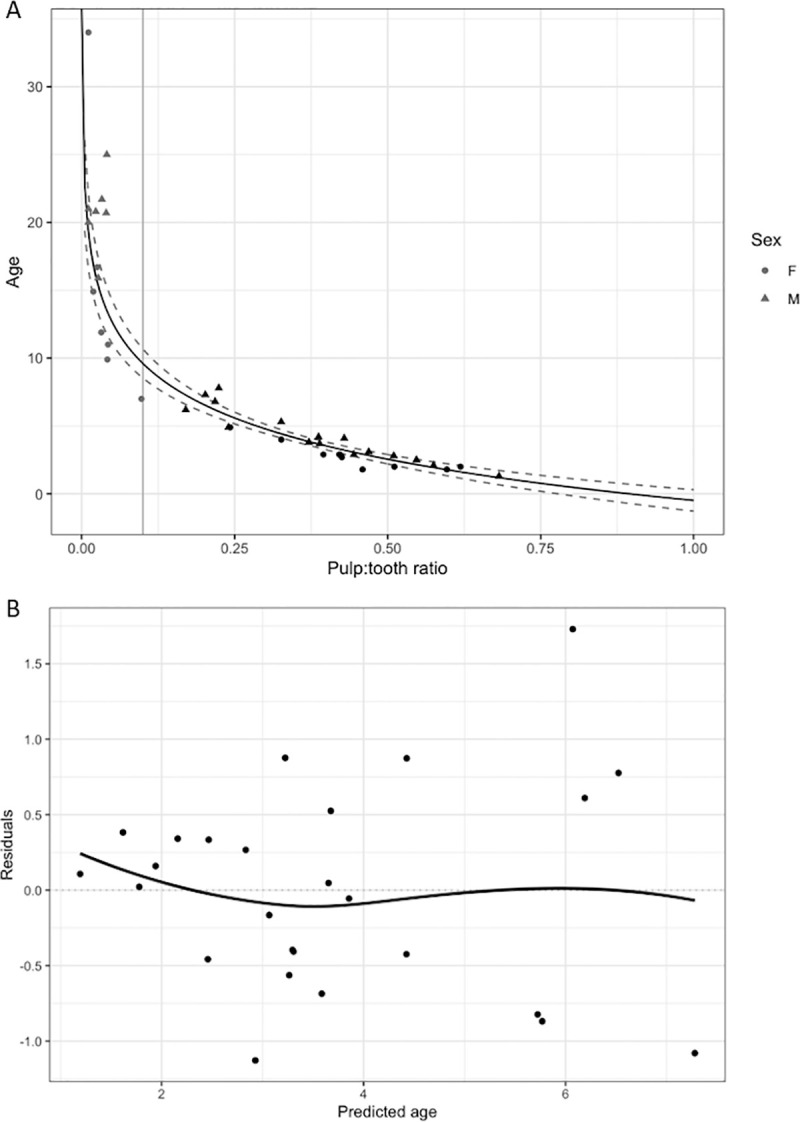
Fit and prediction error of the linear-log regression analysis. (A) Plot of the training dataset used in the linear-log regression analysis to evaluate how well pulp:tooth area ratio can predict age. The points represent individual dolphins with both periapical dental radiographs and life history-based age estimates from SB and BB (n = 40). Due to the low number of samples for pulp:tooth area ratios between 0.04 and 0.17, as well as the wide range of life history-based ages for pulp:tooth area ratios less than 0.04, samples (grey points) with pulp:tooth area ratios < 0.1 (vertical grey line) were excluded. The remaining samples (black points, n = 26) were used to fit a linear-log model of pulp:tooth area ratio against predicted ages (black line, R^2^ = 0.85, confidence limits are given as dashed lines). Due to the low sample size, site or sex were not added as covariates, but there did not appear to be obvious differences between males (triangles) and females (circles), nor between the sites, above the 0.1 threshold. (B) Residual errors for the linear-log model including SB and BB dolphins (n = 26). The increasing variation in residuals with increasing age predictions indicates that the model performs better with larger pulp:tooth area ratios (younger animals).

In total, our model training process included 40 wild dolphins from SB and BB with both periapical radiographs and reliable age estimates (ranging from 1.8 to 34 years old) based on life history (Table 1). As dolphin age increased, pulp:tooth area ratio decreased logarithmically, such that dolphins with pulp:tooth area ratios < 0.1 had life history-based age estimates from 7 to 34 years old (Grey points in Fig 5A) and only one out of 14 (7%) of the dolphins with a pulp:tooth area ratio < 0.1 had a life history age less than 10 years old (S1 Table). Thus, a criterion was applied for our training data set that the pulp area could not be less than 10% of the tooth area (a 0.1 pulp:tooth area ratio) (Table 2). The remaining 26 individual dolphins (with pulp:tooth area ratios ≥ 0.1) were used to fit predictive models. First, a GAM using a gamma distribution and log link function demonstrated that the pulp:tooth area ratio explained 87.2% of the deviance in life history-based age estimates (adjusted R^2^ = 0.85, CV = 16.4%). As the effective degrees of freedom was 1, a linear-log regression analysis was used to test if the pulp:tooth area ratio could predict dolphin age. The results of the regression were, as expected, similar to the GAM, and indicated that pulp:tooth area ratio explained much of the variance in the life history-based age estimates (R^2^ = 0.85, F (1,24) = 138.83). Pulp:tooth area ratio was a strong predictor of age, as determined by life history, (p < 0.00; Fig 5A) based on the equation:

y=−0.4844−4.3816*log(x) (1)

**Table 2:** Sample sizes for each age determination technique by sample group for pulp:tooth area ratio ≥ 0.1.

Group	Dental Radiograph	Male	Female	ND	GLG Age	LH Age	Length (cm)
**BB**	68	40	28	0	27	9	177–267
**MS**	6	6	0	0	6	0	202–234
**SB**	17	7	10	0	0	17	178–238
**Stranded BB**	10	5	2	3	10	0	120–218

BB: Barataria Bay, MS: Mississippi Sound, SB: Sarasota Bay, GLG: Dentinal growth layer group analysis, LH: Observational life history, ND: Data on sex not available.

The prediction error, reported using root mean squared error, as determined by leave-one-out cross-validation was 0.75 years. The variance increased as the predicted age increased (Fig 5B).

**Fig 6:**
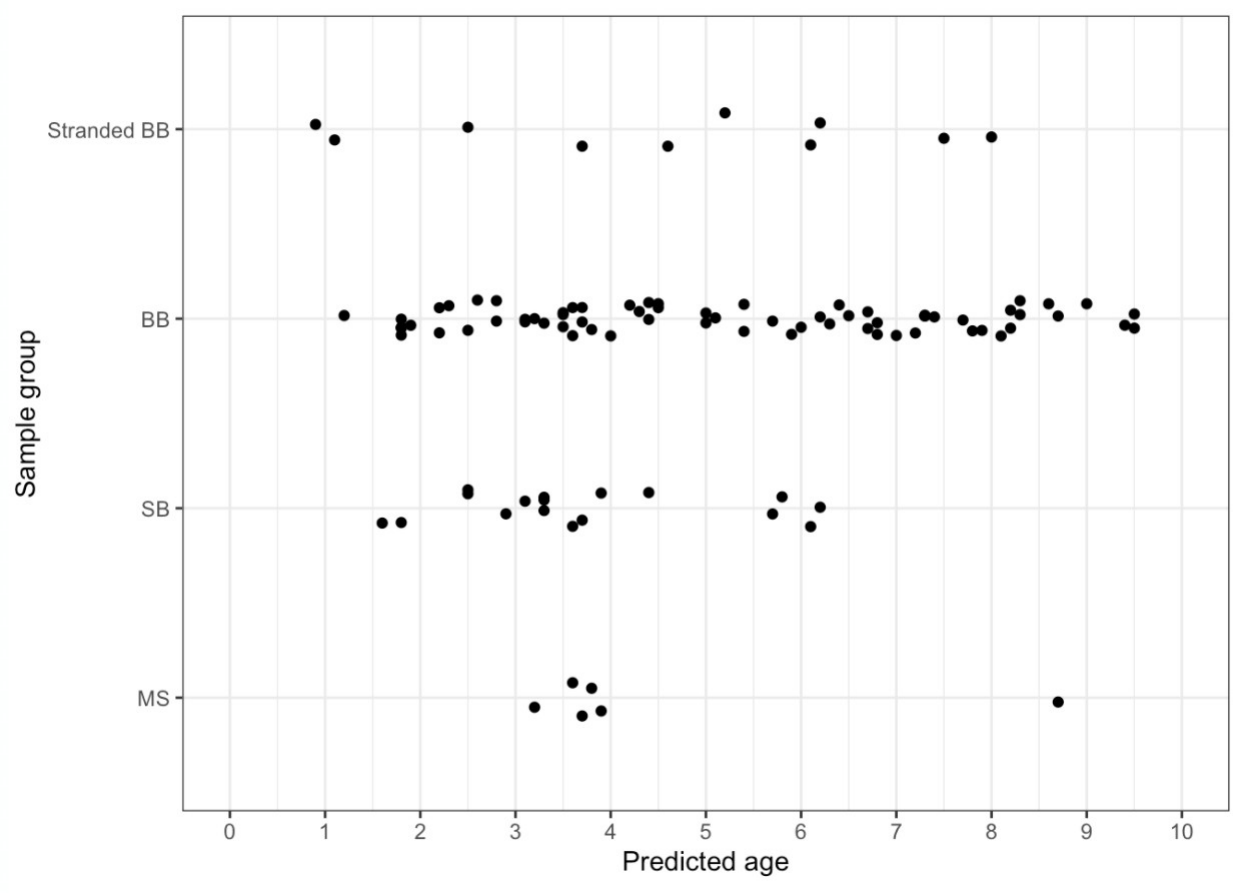
Predicted ages of wild dolphins using periapical dental radiography. Using the linear-log model, the pulp:tooth area ratio was used to predict ages for 91 living dolphins (from health assessments in SB, BB, and MS) and 10 dead, stranded dolphins (from BB). Age distributions should not be interpreted as representative for the populations in question, as the study design for each health assessment, sample availability and model criterion (pulp:tooth area ratio ≥ 0.1) biased the data set.

**Fig 7:**
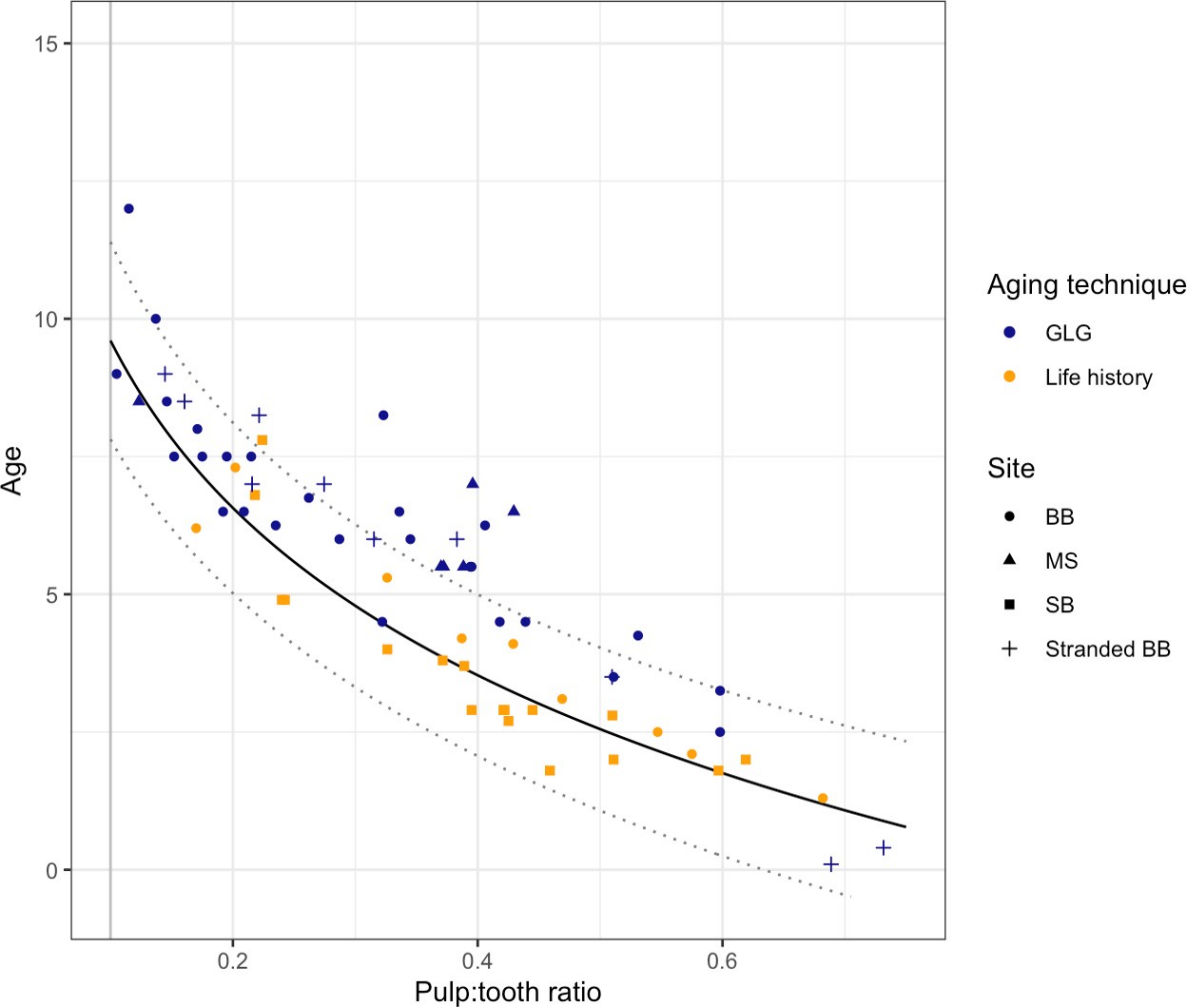
Plot of the linear-log model based on periapical dental radiographs compared to samples with tooth GLG and life history age estimates. Orange points represent the training data set with life history-based age estimates; blue points represent samples without life history information, but with both tooth GLG-based age estimates and periapical dental radiographs. The data set includes both wild, living dolphins (from BB [circles], MS [triangles], and SB [squares]) and dead, stranded dolphins (crosses). The model (black solid line with prediction limits given as dotted lines) consistently predicts lower ages than the GLG-based age estimates (RMSPE = 1.65 years, compared to RMSE = 0.75 for the life history-based age estimates). Data are only shown for samples with a pulp:tooth area ratio ≥ 0.1 (vertical grey line).

Of the original 204 dolphins with periapical radiographs, approximately half (n = 101) had pulp:tooth area ratios < 0.1. Therefore, the linear-log model was used to predict ages based on the pulp:tooth area ratio for these 103 dolphins (Fig 6, S1 Table). The periapical radiography age classes disagreed with: the life history-based age classes in three cases (out of 26, 11.5%—three dolphins classified by life history as 2 years old, were estimated as sub-adults using dental radiographs based on estimated ages of 2.2, 2.5 and 2.9 years old), the GLG-based age classes in four cases (out of 43, 9.3%—two dolphins classified by GLG as adults (10 and 12 years old) were estimated as sub-adults using dental radiographs based on estimated ages of 8.2 and 9 years old, respectively, and two dolphins classified by GLG as sub-adults (2.5 and 3.25 years old) were estimated as calves using dental radiographs based on estimated ages of 1.8 and 1.8 years old, respectively), and the length-based age classes in 17 cases (out of 101, 16.8%—five dolphins classified by length as adults were estimated as sub-adults using dental radiographs based on estimated ages of 9.4, 9.5, 8.6, 9.5 and 3.7 years old, respectively; 10 dolphins classified by length as calves were estimated as sub-adults using dental radiographs based on estimated ages of 2.5 to 3.7 years old; two dolphins classified by length as sub-adults were estimated as calves using dental radiographs based on estimated ages of 1.8 and 1.9 years old, respectively). GLG estimates were consistently higher than our estimated ages obtained using the fitted linear-log model (n = 43 for dolphins with a pulp:tooth area ratio ≥ 0.1, Fig 7). Approximately one third of the GLG age estimates were greater than the model’s upper prediction limit. The two youngest dolphins in the data set (according to GLG age estimates), which were both dead, stranded dolphins from BB, were notable exceptions. The RMSPE for the dolphins with tooth GLG age estimates was 1.65 years—approximately double that of the RMSE for the life history age estimate training data set. The prediction error was similar between GLG ages from live (n = 94, RMSPE = 1.72) and dead, stranded (n = 10, RMSPE = 1.41) dolphins.

**Fig 8:**
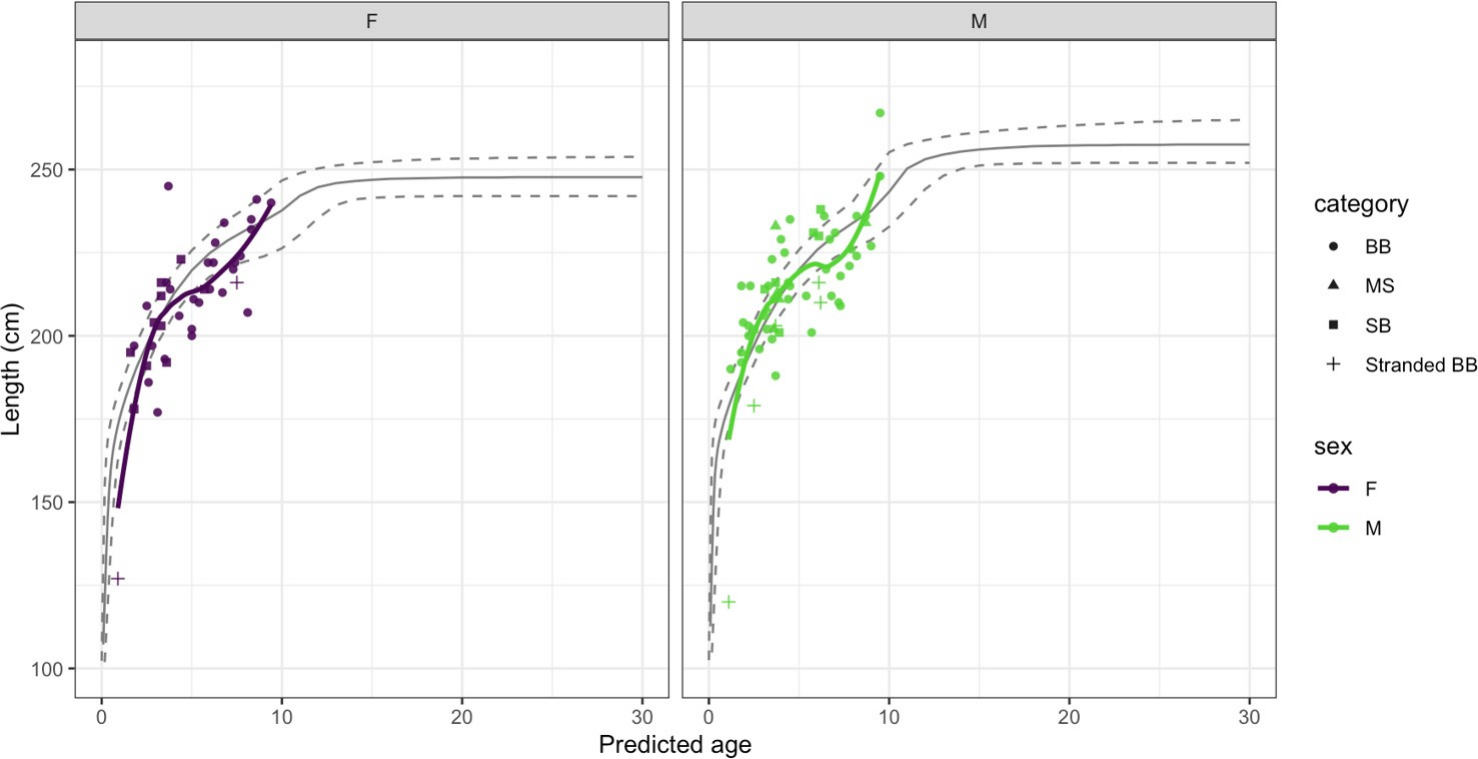
Dental radiography-based age estimates compared to total body length. Data from females are plotted in purple and males are plotted in green. McFee et al. [31] identified three phases of growth in dolphins from Mississippi waters based on GLG age estimates (grey lines represent median fitted values with 95% CI). Although our data set is limited to dolphins generally between the ages of 1–10 years old, the general trend in the periapical age estimates versus length show similar rapid growth of dolphins in this age range.

The predicted ages for males and females were compared to total body length for all 101 dolphins with pulp:tooth area ratios ≥ 0.1 (Fig 8). Although the analysis is limited to dolphins from the ages of approximately one to ten years old, the rate of growth is similar to the three-phase growth curves seen in other populations of dolphins in the southeastern United States [31].

## Discussion

The current standard technique for aging wild dolphins without life history information (observed or inferred birth dates) requires extraction of a vital tooth for analysis of GLG layers [1]. For live dolphins, tooth extraction is an invasive procedure that can be logistically challenging (requiring local anesthesia and specially trained personnel [63]) and must be carefully considered in the context of the study at hand and individual animal welfare. As dentin is continually deposited, the pulp chamber narrows in mammalian teeth. The relationship between tooth and pulpal areas has been used with a widely accepted data analysis method to successfully estimate age in humans and other wildlife species [50, 55 and others]. This method was used here to develop a novel and minimally invasive approach for estimating dolphin age using relatively easy to obtain dental radiographs in multiple field studies of wild dolphins. In addition to being minimally invasive and logistically tractable in the field, dental radiography offers the additional advantage of providing near real-time age estimates for young animals. With a laptop computer, radiographs can be processed and age estimates produced immediately on return to shore at the end of the day, or in some situations onboard the research vessel while still on the water. Such rapid age information can support decisions regarding medical care and/or to prioritize sampling.

Digital periapical radiography was included in 11 bottlenose dolphin health assessment field studies in the Northern Gulf of Mexico from 2013 to 2018. We found the portable veterinary dental x-ray equipment easy to use in the field, both in water and on a processing vessel. Radiation doses associated with dental radiography were very low due to a highly attenuated, very low scatter radiation beam and the use of highly sensitive dental digital phosphor plates, yielding safe methodology for animals and human handlers alike. The procedure was minimally invasive, requiring only brief insertion of the film holder into the dolphin’s mouth for imaging. During the procedure, the primary dolphin handler noted that gentle handling and minimal mouth opening minimized the animal’s reaction to the insertion of the film holder in the mouth, and that although dolphins occasionally moved their heads side-to-side in response, secondary handlers on each side of the head generally could control and minimize such movements.

Radiographic exposure was performed at a fixed kVP of 60 at 2 mA for a standard time of 0.4 seconds. The anatomy of the dolphins’ rostra due to dolphin size did not differ enough to require adjustments of exposure time. The exposure time of 0.4 seconds generated high-quality radiographs and was short enough to avoid blur artifacts associated with slight movements of the dolphin head or wave motion while imaging animals in the water. Once the primary handler and radiographer established a consistent routine after the first set of procedures, dolphin head movements rarely required repeat radiographs in order to obtain high quality images. The bisecting angle allowed the radiographer to collect relatively consistent images that maximized the ability to measure the pulp:tooth area ratio. While slight differences in angle may affect measurements from the image obtained, the use of a ratio of surface areas, instead of direct length or width measurements, ensured consistency across individuals, despite unavoidable, minor variation in angle of radiographic image acquisition.

The fitted GAM and linear-log models using pulp:tooth area ratios were used to estimate age from radiographs obtained from either live or dead, stranded animals. As the pulp cavity closes with age, the accuracy of the age prediction decreases. Unfortunately, the training data set did not include many dolphins with both dental radiographs and life history age estimates (n = 26) to robustly characterize a specific threshold, limiting the ability to perform some analyses (e.g., investigating whether sex or site affects predictions). Thus, based on our visual assessment of where the older dolphins in our data set appeared to fall on the asymptotic portion of a logarithmic curve, pulp:tooth area ratios below 0.1 were excluded (i.e., the pulp area was less than 10% of the tooth area), leaving accurate age estimated to approximately 10 years of age. Future studies with a larger sample size of individuals and potentially integrating multiple types of age estimation techniques could help elucidate how accurate periapical radiography can be for animals over 10 years old, and address our observation that variance increased with predicted age (Fig 3). The youngest individual in our data set with life history information was 1.8 years old. Because temporary capture of dolphins in the U.S. is generally not permitted for younger calves, the only opportunities to identify whether the model predictions are accurate for younger calves will be to compare GLG-based or length-based age estimates from stranded dolphins or dolphins in managed care.

In comparison, the pulp:tooth area ratio has accurately estimated ages to approximately 80 years in humans, which covers most of the typical life expectancy [43, 44, 48–50]. Similarly, Cameriere’s method accurately estimated age throughout the usual lifespan (3–13 years) of the African lion without losing accuracy in older animals [55]. These studies did find that the accuracy of age estimates was impacted by the tooth/teeth selected for analysis, with canines (preferably both upper and lower) performing the best. The dentition of cetaceans is somewhat unique among mammals. They are homodonts (all of their teeth have the same morphology) and monophyodonts (they only have one set of teeth for life), and they have lost precise dental occlusion [72 and references therein]. Thus, there is no concern with error in the aging of young dolphins from deciduous teeth, nor will variability be introduced by aging estimates from different types of teeth. In order to take advantage of the homodonty and increase our confidence in the final pulp:tooth area ratio three teeth per individual were assessed; similarly, using multiple canine teeth has proven to increase accuracy of age determination in other species [50].

Additional studies of wild dolphins, or of dolphins under managed care, with known ages would be useful, since dentin deposition should not be affected by environmental or health issues [38, 39, 44]. The rapid closure of the pulp chamber, assessed radiographically, poses a limitation on this technique in bottlenose dolphins compared to other mammalian species studied [43, 48–55]. The reason for the exuberant dentin deposition and resultant reduction in radiographically visual pulp in bottlenose dolphins at a relatively early age is not currently understood and is an avenue for further research.

Whenever handling is required, minimizing stress to animals is always of primary concern, hence the desire to develop an accurate and minimally-invasive alternative to the technique of aging dolphins by dental GLG analysis. Tooth extraction ordinarily requires that a dolphin be taken aboard a processing vessel and the procedure itself usually takes at least 15–20 min from local anesthesia to completion of the tooth extraction; therefore, some dolphins are not suitable candidates for tooth extraction in the field. The time required to acquire dental radiographs for age determination is much shorter, approximately 2–5 minutes, and can be easily accomplished in the water or on the processing vessel, as needed, thus allowing for dental radiographs for almost all dolphins sampled.

Recently, measurements of skeletal ossification from pectoral flipper radiographs have been reported to successfully age dolphins from managed populations to > 30 years, however, precision decreased in animals > 20 years [9]. Precision of dental radiographic methods are similar in animals < 10 years, while pectoral flipper radiography does appear to be more precise in older dolphins. However, the pectoral flipper radiographic method has not yet been validated in wild dolphins, and in humans, growth plate closures are known to have more pressure from health-related issues, such as nutrition and wear and tear that affect the accuracy of the technique, whereas health issues have not affected dentinal deposition [38, 39, 44]. It is possible that variability in growth plate closures may be significantly greater in these wild populations exposed to more diverse environmental stressors compared to their counterparts under human care. These and other questions will be addressed as more pectoral flipper radiography data are collected and can be directly compared to other age estimation techniques. The primary disadvantages of the pectoral flipper radiographs are the equipment cost and logistical challenges associated with application in a field setting. These include higher radiation exposures to dolphins and handlers, obtaining high quality radiographs in or near the water, and potentially increasing time out of water if radiographs must be performed on a processing vessel due to sea conditions. Minimizing time out of water is a priority during dolphin health assessments, and because many animals are fully processed in-water, the additional flexibility afforded by dental radiography in field settings is advantageous.

Age obtained from life history observations was used to fit the linear-log regression, resulting in a prediction error (RMSE) of about ± nine months (based on leave-one-out cross validation). When our estimates from the model output were compared to GLG age estimates, the RMSPE was about ± 20 months. Usually the GLG age estimates were greater than those from periapical radiography. Interestingly, Hohn et al. [1] note that accessory layers of dentin may occasionally lead to ages that are erroneously older by about two years. Further studies that would include age determination by life history, GLG and periapical radiography in the same animal could help clarify the discrepancies observed here. If there are biases between the GLG and dental radiography age estimates, they seem to be consistent across the age range of our test data set, as the overall three-phase growth rate reported in other studies and based on GLG estimates [31] is consistent with the growth rate suggested by the periapical radiography-based age estimates (Fig 8).

A significant advantage of the radiographic method is the ability to acquire same-day results. Dental radiographs require minimal processing post-image acquisition in the field, compared to the, typically, weeks to months turn-around time for GLG analysis, potentially allowing researchers to conduct a more age-appropriate health assessment in near-real-time. For example, YY7, a dolphin captured during the 2018 BB health assessment, was initially estimated to be two to three years old based on length. However, later that evening, the field radiographer evaluated the pulp:tooth area ratio and estimated YY7’s age as five to six years old. When YY7 was re-sampled the same year, the better estimate of age allowed the veterinary team to complete a more age-appropriate health assessment (e.g., reproductive ultrasound).

**Fig 9:**
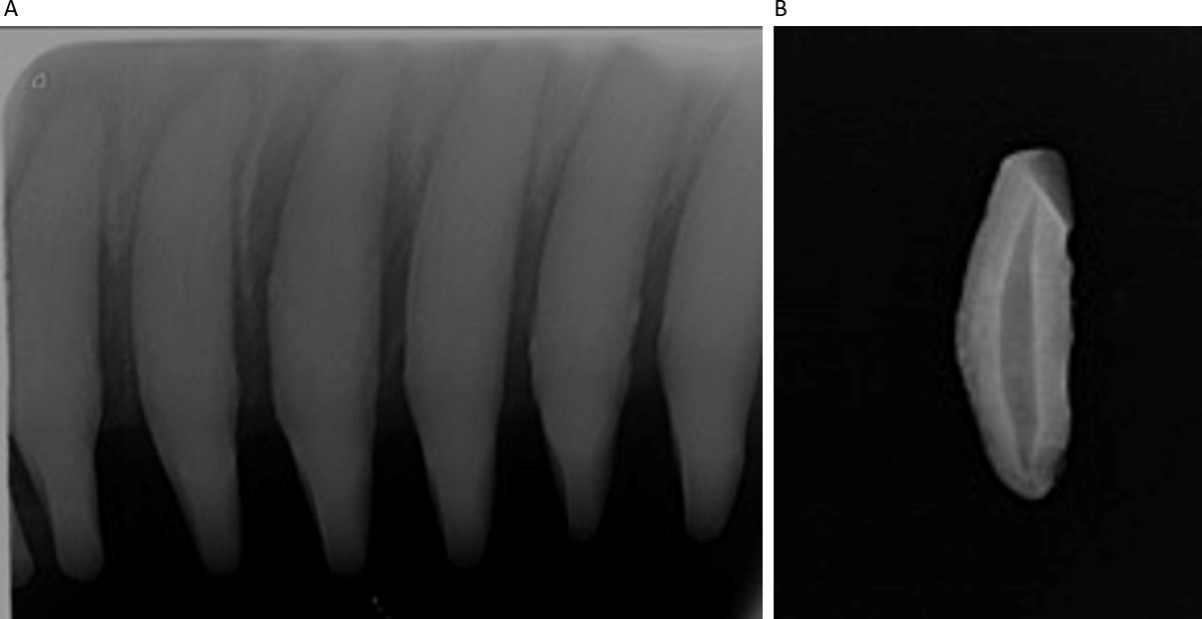
Periapical radiographs of dolphin YN9. Even in cases where the pulp:tooth area ratio is below 0.1 and excluded from age estimation by the model; dental radiographs can help confirm other age estimation techniques. Here, dolphin YN9’s pulp area is very small (A), indicating an older animal. However, radiography shows that the single tooth extracted for GLG-based age estimation is in fact non-vital (B), and therefore likely results in a younger age estimation.

The inclusion of several teeth on a film provides built-in quality control. Since both X-ray analysis and GLG methods rely on the deposition of dentin over time, they require observations from a viable tooth. Several instances were observed where the pulp:tooth area ratio in one of the teeth was obviously greater than surrounding teeth on the captured radiograph image, likely as a result of a dead tooth which ceased to deposit dentin to close the pulp cavity (Fig 4). Such a dead tooth can easily be excluded from the pulp-tooth area ratio analysis using the x-ray method. However, the extraction of a tooth for GLG analysis would not identify such an artifact from a dead tooth and would result in an inaccurate age determination. An example of this occurred with BB dolphin YN9, who had a young GLG age that did not match the animal’s length or known reproductive history. Further, the lack of visible pulp on dental radiography and very low pulp/tooth area ratio suggest that the dolphin was >10 years old, and certainly older than the GLG estimate (7 years). Upon examination, radiographs of the extracted tooth were not similar to radiographic images of the teeth selected for pulp:tooth area ratio analysis, indicating the extracted tooth was a non-vital tooth (Fig 9).

The results presented here demonstrate the use of digital periapical radiography to estimate age using pulp:tooth area ratios in wild bottlenose dolphin populations, especially for younger dolphins. To our knowledge, this is only the second wildlife species in which Cameriere’s method of age estimation has been applied [55]. The dental pulp:tooth area ratio technique is valuable as presented in this data set in animals under 10 years. The technique could be refined in future studies by additional dental radiography of known life history aged animals (wild or managed care), especially those aged 8–20 years, to expand the range of animals accurately aged by this technique. It still has value in older animals to confirm age class and estimates will be more informative than length alone once asymptotic length is reached. As other techniques evolve for field use, they may be valuable in aging older animals over the age of 10.

This dental radiographic method is a strong predictor of age, particularly for younger dolphins, as determined by longitudinal monitoring of individually identifiable animals (life history) and performs similarly to widely used dentinal GLG analysis. Dental radiology is significantly less invasive, demonstrates ease of use during in-water field work, has a simplified data analysis with a built-in quality control, requires less holding time for the dolphin and can produce same day estimates as compared to tooth extraction or other currently validated techniques apart from life history information. This method can greatly expand the number of dolphins accurately aged when temperament, health or field conditions are not amenable to allow for tooth extraction or other techniques. Additionally, it is a rapid method that is highly applicable to use in capture-release health assessments, live stranding response and rehabilitation settings and the equipment required is not cost-prohibitive. Wide application of this method to age bottlenose dolphins will be invaluable to research, conservation and management efforts.

## Supporting information

S1 Table Sample demographics. Demographic data for all samples including, sample location, sex, length and age estimates and associated age class from pulp:tooth area ratio, GLG or life history data. Calculated pulp:tooth area ratios are also listed.
(XLSX)

## References

[1] Hohn AA, Scott MD, Wells RS, Sweeney JC, Irvine AB. Growth layers in teeth from known-age, free-ranging bottlenose dolphins. Mar Mamm Sci. 1989;5(4):315–42.

[2] Read AJ, Wells RS, Hohn AA, Scott MD. Patterns of growth in wild bottlenose dolphins, *Tursiops truncatus*. J Zoo. 1993;231(1):107–23.

[3] Ferrero RC, Walker WA. Age, growth, and reproductive patterns of Dall's porpoise (*Phocoenoide dalli*) in the central North Pacific Ocean. Mar Mamm Sci. 1999;15(2):273–313.

[4] McFee WE, Adams JD, Fair PA, Bossart GD. Age distribution and growth of two bottlenose dolphin (*Tursiops truncatus*) populations from capture-release studies in the southeastern United States. Aquat Mamm. 2012;38(1):17–30.

[5] Myrick AC Jr, Shallenberger EW, Kang I, MacKay DB. Calibration of dental layers in seven captive Hawaiian spinner dolphins, *Stenella logirostris*, based on tetracycline labeling. Fishery Bulletin. 1984;82(1):207–25.

[6] Oosthuizen WH, Bester MN. Comparison of age determination techniques for known-age cape fur seals. S Afr J Zool. 1997;32(4):106–1.

[7] Marmontel M, Nhea TJ, Kochman HI, Humphrey SR. Age determination in manatees using growth-layer-group counts in bone. Mar Mamm Sci. 1996;12(1):54–88.

[8] Sensor JD, George JC, Clementz MT, Lovano DM, Waugh DA, Givens GH, et al Age estimation in bowhead whales using tympanic bulla histology and baleen isotopes. Mar Mamm Sci. 2018;34(2):347–64.

[9] Barratclough A, Sanz-Requena R, Marti-Bonmati L, Schmitt TL, Jensen E, García-Párraga D. Radiographic assessment of pectoral flipper bone maturation in bottlenose dolphins (*Tursiops truncatus*), as a novel technique to accurately estimate chronological age. PLoS ONE. 2019;14(9):e0222722 10.1371/journal.pone.0222722 31557197 PMC6762177

[10] Powell JWB, Duffield DA, Kaufman JJ, McFee WE. Bone density cannot accurately predict age in the common bottlenose dolphin, *Tursiops truncatus*. Mar Mamm Sci. 2019;35(4):1597–602.

[11] Butti C, Corain L, Cozzi B, Podestà M, Pirone A, Affronte M, et al Age estimation in the Mediterranean bottlenose dolphin *Tursiops truncatus* (Montagu 1821) by bone density of the thoracic limb. J Anat. 2007;211(5):639–46. 10.1111/j.1469-7580.2007.00805.x 17850286 PMC2375788

[12] Guglielmini C, Zotti A, Bernardini D, Pietra M, Podestá M, Cozzi B. Bone density of the arm and forearm as an age indicator in specimens of stranded striped dolphins (stenella coeruleoalba). Anat Rec. 2002;267(3):225–30. 10.1002/ar.10107 12115272

[13] Loch C, Schwass DR, Kieser JA, Fordyce RE. Use of micro-computed tomography for dental studies in modern and fossil odontocetes: Potential applications and limitations. NAMMCO Sci Publ. 2013;10(0).

[14] Kastelle CR, Shelden KEW, Kimura DK. Age determination of mysticete whales using 210pb/226ra disequilibria. Can J Zool. 2003;81(1):21–32.

[15] Sukhovskaya LI, Klevezal GA, Borisov VI, Lagerev SJ. Use of bone layers to determine age in minke whales. Acta Theriol. 1985;30(17):275–86.

[16] George JC, Bada J, Zeh J, Scott L, Brown SE, O'Hara T, et al Age and growth estimates of bowhead whales (*Balaena mysticetus*) via aspartic acid racemization. Can J Zool. 1999;77(4):571–80.

[17] Wetzel D, Reynolds J, Mercurio P, Givens G, Pulster E, George J. Age estimation for bowhead whales, *Balaena mysticetus*, using aspartic acid racemization with enhanced hydrolysis and derivatization procedures 2014.

[18] Boye TK, Garde E, Nielsen J, Hedeholm R, Olsen J, Simon M. Estimating the age of West Greenland humpback whales through aspartic acid racemization and eye lens bomb radiocarbon methods. Frontiers in Marine Science. 2020;6(811).

[19] Schell DM, Saupe SM, Haubenstock N. Bowhead whale (*Balaena mysticetus*) growth and feeding as estimated by δ13c techniques. Marine Biology. 1989;103(4):433–43.

[20] Schell DM, Saupe SM, Haubenstock N. Natural isotope abundances in bowhead whale (*Balaena mysticetus*) baleen: Markers of aging and habitat usage Stable Isotopes in Ecological Research: Springer New York; 1989 p. 260–9.

[21] Campana SE, Stewart REA. Bomb dating, age validation and quality control of age determinations of monodontids and other marine mammals. NAMMCO Sci Publ. 2014;10(0).

[22] Garde E, Frie AK, Dunshea G, Hansen SH, Kovacs KM, Lydersen C. Harp seal ageing techniques—teeth, aspartic acid racemization, and telomere sequence analysis. J Mammal. 2010;91(6):1365–74.

[23] Izzo C, Hamer DJ, Bertozzi T, Donnellan SC, Gillanders BM. Telomere length and age in pinnipeds: The endangered Australian sea lion as a case study. Mar Mamm Sci. 2011;27(4):841–51.

[24] Dunshea G, Duffield D, Gales N, Hindell M, Wells RS, Jarman SN. Telomeres as age markers in vertebrate molecular ecology. Mol Ecol Resour. 2011;11(2):225–35. 10.1111/j.1755-0998.2010.02976.x 21429128

[25] Beal AP, Kiszka JJ, Wells RS, Eirin-Lopez JM. The bottlenose dolphin epigenetic aging tool (BEAT): A molecular age estimation tool for small cetaceans. Frontiers in Marine Science. 2019;6(561).

[26] Koopman HN, Iverson SJ, Gaskin DE. Stratification and age-related differences in blubber fatty acids of the male harbour porpoise (*Phocoena phocoena*). J Comp Physiol B Biochem Syst Environ Physiol. 1996;165(8):628–39. 10.1007/BF00301131 8882509

[27] Herman DP, Matkin CO, Ylitalo GM, Durban JW, Hanson MB, Dahlheim ME, et al Assessing age distributions of killer whale *Orcinus orca* populations from the composition of endogenous fatty acids in their outer blubber layers. Mar Ecol Prog Ser. 2008;372:289–302.

[28] Herman DP, Ylitalo GM, Robbins J, Straley JM, Gabriele CM, Clapham PJ, et al Age determination of humpback whales *Megaptera novaeangliae* through blubber fatty acid compositions of biopsy samples. Mar Ecol Prog Ser. 2009;392:277–93.

[29] Marcoux M, Lesage V, Thiemann GW, Iverson SJ, Ferguson SH. Age estimation of belugas, *Delphinapterus leucas*, using fatty acid composition: A promising method. Mar Mamm Sci. 2015;31(3):944–62.

[30] Schwarz LK, Runge MC. Hierarchical Bayesian analysis to incorporate age uncertainty in growth curve analysis and estimates of age from length: Florida manatee (*Trichechus manatus*) carcasses. Can J Fish Aquat Sci. 2009;66(10):1775–89.

[31] McFee WE, Schwacke JH, Stolen MK, Mullin KD, Schwacke LH. Investigation of growth phases for bottlenose dolphins using a Bayesian modeling approach. Mar Mamm Sci. 2010;26(1):67–85.

[32] Wells RS. Social structure and life history of bottlenose dolphins near Sarasota Bay, Florida: Insights from four decades and five generations In: Yamagiwa J, Karczmarski L, editors. Primates and Cetaceans: Field Research and Conservation of Complex Mammalian Societies. Tokyo: Springer Japan; 2014 p. 149–72.

[33] Hohn AA. Age determination and age related factors in the teeth of western North Atlantic bottlenose dolphins. Scientific Reports of the Whales Research Institute. 1980;32:39–66.

[34] Scott M, Wells R, Irvine AB. Long-term studies of bottlenose dolphins in Florida. J Mar Biol Assoc U K. 1996;I.B.I Reports:73–81.

[35] Hohn AA, Fernandez S. Biases in dolphin age structure due to age estimation technique. Mar Mamm Sci. 1999;15(4):1124–32.

[36] Stolen MK, Barlow J. A model life table for bottlenose dolphins (*Tursiops truncatus*) from the Indian River Lagoon sysytem, Florida, U.S.A. Mar Mamm Sci. 2003;19(4):630–49.

[37] Hohn AA. Age estimation In: Perrin WF, Würsig B, Thewissen JGM, editors. Encyclopedia of Marine Mammals (Second Edition). London: Academic Press; 2009 p. 11–7.

[38] Morse DR. Age-related changes of the dental pulp complex and their relationship to systemic aging. Oral Surg Oral Med Oral Pathol. 1991;72(6):721–45. 10.1016/0030-4220(91)90019-9 1812456

[39] Schmeling A, Geserick G, Reisinger W, Olze A. Age estimation. Forensic Sci Int. 2007;165(2):178–81. 16782291 10.1016/j.forsciint.2006.05.016

[40] Perrin WF, Myrick ACJ, editors. Age determination of toothed whales and sirenians. International Conference on Determining Age of Odontocete Cetaceans (and Sirenians); 1980 Sep 5–19, 1978; La Jolla, CA: Cambridge (UK) IWC.

[41] Lockyer C. A review of factors involved in zonation in odontocete teeth, and an investigation of the likely impact of environment factors and major life events on harbour porpoise tooth structure. Reports of the International Whaling Commission. 1995:511–29.

[42] Dellabianca NA, Hohn AA, Goodall RNP, Pousa JL, MacLeod CD, Lima M. Influence of climate oscillations on dentinal deposition in teeth of Commerson's dolphin. Glob Change Biol. 2012;18(8):2477–86.

[43] Cameriere R, Brogi G, Ferrante L, Mirtella D, Vultaggio C, Cingolani M, et al Reliability in age determination by pulp/tooth ratio in upper canines in skeletal remains. J Forensic Sci. 2006;51(4):861–4. 10.1111/j.1556-4029.2006.00159.x 16882230

[44] Cameriere R, Cunha E, Sassaroli E, Nuzzolese E, Ferrante L. Age estimation by pulp/tooth area ratio in canines: Study of a Portuguese sample to test Cameriere's method. Forensic Sci Int. 2009;193(1):128.e1–.e6. 10.1016/j.forsciint.2009.09.011 19854595

[45] Kvaal SI, Kolltveit KM, Thomsen IO, Solheim T. Age estimation of adults from dental radiographs. Forensic Sci Int. 1995;74(3):175–85. 10.1016/0379-0738(95)01760-g 7557754

[46] Philippas GG. Influence of occlusal wear and age on formation of dentin and size of pulp chamber. J Dent Res. 1961;40(6):1186–98.

[47] Solheim T. A new method for dental age estimation in adults. Forensic Sci Int. 1993;59(2):137–47. 10.1016/0379-0738(93)90152-z 8330806

[48] Cameriere R, Ferrante L, Cingolani M. Variations in pulp/tooth area ratio as an indicator of age: a preliminary study. Journal of forensic sciences. 2004;49(2):317–9. 15027553

[49] Cameriere R, Ferrante L, Belcastro MG, Bonfiglioli B, Rastelli E, Cingolani M. Age estimation by pulp/tooth ratio in canines by peri-apical x-rays. J Forensic Sci. 2007;52(1):166–70. 10.1111/j.1556-4029.2006.00336.x 17209930

[50] Cameriere R, Ferrante L, Belcastro MG, Bonfiglioli B, Rastelli E, Cingolani M. Age estimation by pulp/tooth ratio in canines by mesial and vestibular peri-apical x-rays. J Forensic Sci. 2007;52(5):1151–5. 10.1111/j.1556-4029.2007.00530.x 17680998

[51] De Luca S, Alemán I, Bertoldi F, Ferrante L, Mastrangelo P, Cingolani M, et al Age estimation by tooth/pulp ratio in canines by peri-apical x-rays: Reliability in age determination of Spanish and Italian medieval skeletal remains. J Archaeol Sci. 2010;37(12):3048–58.

[52] De Luca S, Bautista J, Alemán I, Cameriere R. Age-at-death estimation by pulp/tooth area ratio in canines: Study of a 20th-century Mexican sample of prisoners to test Cameriere’s method. J Forensic Sci. 2011;56(5):1302–9. 10.1111/j.1556-4029.2011.01784.x 21496018

[53] Jeevan MB, Kale AD, Angadi PV, Hallikerimath S. Age estimation by pulp/tooth area ratio in canines: Cameriere's method assessed in an Indian sample using radiovisiography. Forensic Sci Int. 2011;204(1):209.e1–.e5. 20869824 10.1016/j.forsciint.2010.08.017

[54] Zaher JF, Fawzy IA, Habib SR, Ali MM. Age estimation from pulp/tooth area ratio in maxillary incisors among Egyptians using dental radiographic images. J Forensic Leg Med. 2011;18(2):62–5. 10.1016/j.jflm.2010.12.004 21315299

[55] White PA, Ikanda D, Ferrante L, Chardonnet P, Mesochina P, Cameriere R. Age estimation of African lions *Panthera leo* by ratio of tooth areas. PLoS ONE. 2016;11(4):e0153648 10.1371/journal.pone.0153648 27089506 PMC4835051

[56] Wells RS, Rhinehart HL, Hansen LJ, Sweeney JC, Townsend FI, Stone R, et al Bottlenose dolphins as marine ecosystem sentinels: Developing a health monitoring system. EcoHealth. 2004;1(3):246–54.

[57] Balmer B, Zolman E, Rowles T, Smith C, Townsend F, Fauquier D, et al Ranging patterns, spatial overlap, and association with dolphin morbillivirus exposure in common bottlenose dolphins (*Tursiops truncatus*) along the Georgia, USA coast. Ecol Evol. 2018;8(24):12890–904. 10.1002/ece3.4727 30619591 PMC6308875

[58] Barratclough A, Wells RS, Schwacke LH, Rowles TK, Gomez FM, Fauquier DA, et al Health assessments of common bottlenose dolphins (*Tursiops truncatus*): Past, present, and potential conservation applications. Front Vet Sci. 2019;6(444). 10.3389/fvets.2019.00444 PMC6923228 31921905

[59] Schwacke LH, Smith CR, Townsend FI, Wells RS, Hart LB, Balmer BC, et al Health of common bottlenose dolphins (*Tursiops truncatus*) in Barataria Bay, Louisiana, following the D*eepwater Horizon* oil spill. Environ Sci Technol. 2014;48(1):93–103. 10.1021/es403610f 24350796

[60] Smith CR, Rowles TK, Hart LB, Townsend FI, Wells RS, Zolman ES, et al Slow recovery of Barataria Bay dolphin health following the *Deepwater Horizon* oil spill (2013–2014), with evidence of persistent lung disease and impaired stress response. Endanger Spec Res. 2017;33:127–42.

[61] Cockcroft VG, Ross GJB. Observations on the early development of a captive bottlenose dolphin calf In: Leatherwood S, Reeves RR, editors. The Bottlenose Dolphin. San Diego: Academic Press; 1990 p. 461–78.

[62] Kastelein RA, Dokter T, Zwart P. The suckling of a bottlenose dolphin calf (*Tursiops truncatus*) by a foster mother, and information on transverse birth bands. Aquat Mamm. 1990;16(3):134–8.

[63] Townsend FI, Smith CR, Rowles TK. Health assessment in bottlenose dolphins in capture-release studies In: Gulland FMD, Dierauf LA, Whitman KL, editors. CRC Handbook of Marine Mammal Medicine. Third ecition ed. Boca Raton, FL: Taylor and Francis; 2018 p. 823–33.

[64] Schwacke LH, Twiner MJ, De Guise S, Balmer BC, Wells RS, Townsend FI, et al Eosinophilia and Biotoxin Exposure in Bottlenose Dolphins (Tursiops Truncatus) from a Coastal Area Impacted by Repeated Mortality Events. Environ Res. 2010;110(6):548–55. 10.1016/j.envres.2010.05.003 20537621

[65] R Core Team. R: A language and environment for statistical computing. Vienna, Austria: R Foundation for Statistical Computing; 2018.

[66] RStudio Team. Rstudio: Integrated development for R. Boston, MA: RStudio, Inc; 2016.

[67] Wickham H, Averick M, Bryan J, Chang W, D'Agostino McGowan L, François R, et al Welcome to the Tidyverse. Journal of Open Source Software. 2019;4(43):1686.

[68] Wood S. Generalized additive models: An introduction with R. 2 ed: Chapman and Hall/CRC; 2017. 391 p.

[69] Wood SN. Fast stable restricted maximum likelihood and marginal likelihood estimation of semiparametric generalized linear models. J R Stat Soc Series B Stat Methodol. 2011;73(1):3–36.

[70] Venables B, Ripley B. Modern applied statistics with S. 4th edition ed. New York, NY: Springer; 2002.

[71] Kuhn M. caret: Classification and Regression Training. R package version 6.0–86. https://CRAN.R-project.org/package=caret2020.

[72] Armfield BA, Zheng Z, Bajpai S, Vinyard CJ, Thewissen J. Development and evolution of the unique cetacean dentition. PeerJ. 2013;1:e24–e. 10.7717/peerj.24 23638359 PMC3628747

